# Combined Docking with Classical Force Field and Quantum Chemical Semiempirical Method PM7

**DOI:** 10.1155/2017/7167691

**Published:** 2017-01-16

**Authors:** A. V. Sulimov, D. C. Kutov, E. V. Katkova, V. B. Sulimov

**Affiliations:** ^1^Dimonta Ltd., Nagornaya Str. 15, Building 8, Moscow 117186, Russia; ^2^Research Computer Center (NIVC), M.V. Lomonosov Moscow State University (MGU), Leninskiye Gory 1, Building 4, Moscow 119991, Russia

## Abstract

Results of the combined use of the classical force field and the recent quantum chemical PM7 method for docking are presented. Initially the gridless docking of a flexible low molecular weight ligand into the rigid target protein is performed with the energy function calculated in the MMFF94 force field with implicit water solvent in the PCM model. Among several hundred thousand local minima, which are found in the docking procedure, about eight thousand lowest energy minima are chosen and then energies of these minima are recalculated with the recent quantum chemical semiempirical PM7 method. This procedure is applied to 16 test complexes with different proteins and ligands. For almost all test complexes such energy recalculation results in the global energy minimum configuration corresponding to the ligand pose near the native ligand position in the crystalized protein-ligand complex. A significant improvement of the ligand positioning accuracy comparing with MMFF94 energy calculations is demonstrated.

## 1. Introduction

Reliable predictions of the target protein inhibition by a low molecular weight ligand is defined by the accuracy of docking programs which carry out positioning of the ligand in the active site of the target protein and calculate the protein-ligand binding free energy. Obviously, the positioning accuracy and the accuracy of the binding energy calculation are closely linked: faulty positioning cannot result in the high accuracy of the binding energy calculation based on the found ligand poses. The docking accuracy depends on many factors: the docking algorithm, the method of calculation of the molecular system free energy, the presence or absence of solvent, the protein model, and so forth. The docking algorithm is based on the docking paradigm, which assumes that the native ligand position in the active site of the target protein is close to the global energy minimum of the protein-ligand system. The search space dimension for the molecular system consisting of a flexible drug-like ligand and a rigid target protein is usually 10–15 when the system has 10–15 degrees of freedom. So, the search for the global energy minimum (or the whole range of low-energy minima) in such multidimensional space is not an easy problem. Various docking programs apply different methods of the global minimum search on the multidimensional energy surface and solve this problem with different accuracy. In docking programs the energy of the protein-ligand system is calculated using one of the force fields. The correctness of the docking paradigm [1] is not obvious in this case, since most force fields were created mainly to describe the molecular geometry but not intermolecular interactions playing the crucial role in ligand-protein binding.

Nevertheless, recent studies [1–3] show that docking paradigm is valid with the MMFF94 force field [4] for some protein-ligand complexes and solvent accounting plays an important role. These studies also revealed a trend towards an improvement of docking positioning when the protein-ligand energy calculated with the MMFF94 force field was replaced by the energy calculated with the recent PM7 quantum chemical semiempirical method [5]. Similarly, the semiempirical filter [6], SQM/COSMO, on the base of the PM6-D3H4X method [7, 8] together with the COSMO solvent model [9] has demonstrated recently the best performance in docking and subsequent energy calculations comparing with eight scoring functions constructed on the base of classical force fields. Application of semiempirical quantum chemical methods to biological and drug design problems becomes popular in recent years [10–16] (see also a review [17] and references in [6]), but usually they are used at the postdocking stage of calculations because quantum chemical docking demands much more computational resources than force fields based docking.

In this paper we present the results of examination of the appropriateness of the PM7 method use for docking: can the replacement of the force field by the quantum chemical method PM7 improve the docking positioning accuracy?

The feasibility of docking with PM7 protein-ligand energy calculations is demonstrated. It is shown that the employment of the PM7 method with the implicit solvent model improves significantly the accuracy of the ligand positioning and as a result the docking paradigm becomes true for most of the investigated complexes.

## 2. Methods

### 2.1. New Semiempirical Quantum Chemical Method PM7

Currently there are two main approaches for atomistic modeling of molecular systems: methods of classical force fields and quantum chemical methods. The application of nonempirical (ab initio) quantum chemical methods has limitations on the size of molecular systems from a few dozen to a few hundred atoms. Therefore, only semiempirical quantum chemical methods can be applied to calculate the energy of the protein-ligand system (many thousands of atoms). Although these methods are worse than ab initio ones, they are considerably faster and they undoubtedly excel any classical force field in accuracy and universality. Semiempirical methods had been widely used in the 1990s with their development and creation of appropriate programs, for example, MOPAC [18]. These methods significantly simplify the calculation of multicenter two-electron integrals due to neglecting a lot of these integrals, due to the approximate calculation of other integrals and by introducing empirically selected parameters for the better agreement between calculated molecular characteristics and experimental data. Despite a considerable improvement of the accuracy of these methods over the past few decades, their main drawback until recently was a poor description of dispersion interactions and hydrogen bonds [19]. Recently, however, the new РМ6 [20, 21] and PM7 [5] methods were developed on the base of the NDDO (Neglect of Diatomic Differential Overlap) approximation. Ideas that have been successfully used previously in DFT methods [22, 23] to describe dispersion interactions and hydrogen bonds are implemented in PM6 and PM7 methods. Moreover, the PM7 method includes the description of dispersion interactions and hydrogen and halogen bonding at the parameterization stage which has been performed on an extremely large set of molecular data.

Last versions of the MOPAC program [24] also include the MOZYME module [25], where the usual LCAO (Linear Combination of Atomic Orbitals) approximation is replaced with the localized molecular orbitals (LMO) method. This leads to a linear dependence of the computing time on the size of the molecular system and allows calculations of molecular systems up to 18,000 atoms. Thus, MOZYME makes it possible to apply the PM7 method (as well as PM6 methods) to the calculation of protein-ligand complexes characteristics; for example, see [26].

The PM7 method stands in the row together with several modifications of the PM6 method: PM6-DH+, PM6-DH2(X), and PM6-D3H4(X). All of them describe noncovalent interactions on the same ground, they have their own peculiarities of parameterization and demonstrate comparable accuracies, and the PM7 method is not the best as it has been demonstrated in detailed validation [27]. However, in the present study we choose the PM7 method as “the most robust tool among the semiempirical methods, suitable for modeling a wide range of species” [27].

The PM7 calculation of the protein-ligand complex with fixed geometry takes only a few minutes. The optimization of the ligand geometry in the fixed protein can take from several hours to several days depending on the ligand size. At the same time docking often requires many millions of energy calculations. Bearing this in mind, it is clear that it is impossible so far to perform the docking based on the protein-ligand energy calculation with the PM7 method. However, since supercomputers power grows up rapidly, it seems reasonable to evaluate the effectiveness of docking based on the quantum chemical PM7 method. For this purpose we use a hybrid procedure, quasi-docking, consisting of the following steps.

### 2.2. Quantum Chemical Quasi-Docking

This procedure performs direct (gridless) generalized docking in the frame of the MMFF94 force field and keeps a fairly wide spectrum of low-energy minima including the global minimum and then performs subsequent recalculation of these minima energies with the PM7 method. Previous studies have shown [1] that local minima of protein-ligand complexes do not differ too much from each other in space (i.e., the respective ligand poses are close to each other) for various target energy functions: either to calculate energy with the MMFF94 force field or with the PM7 method in vacuum or in solvent. The transition from one energy function to the other one changes insignificantly the positions of minima (after the respective optimization), but the minima energies can vary considerably. The minima set of the given protein-ligand complex with minima energies calculated by a given target function can be sorted by their energy in ascending order and this minima's ranking can change significantly with the change of the protein-ligand energy function. So, the global minimum calculated with one energy function can rise much higher than many other minima when minima energies are recalculated with another energy function. Thus, for various energy functions of a given protein-ligand complex the multidimensional energy surface landscape remains almost the same. Minima positions shift only slightly, but their relative depths can change significantly. This implies that when we find all (or almost all) minima with one energy function, we do not need to carry out again time-consuming docking to find minima for another energy function. We can simply recalculate energies of the previously found minima with the new energy function with or without the respective local optimization. We use this idea in the present work to perform the quasi-docking procedure for test set of complexes from [1]. At first we have found a very large number (8192) of low-energy minima using the generalized direct docking program FLM [1, 2] when the energy target function is calculated with the MMFF94 force field [4] and with the PCM implicit solvent model [28, 29]. Then these minima have been recalculated with the PM7 method [5] and the COSMO solvent model [9] using the MOPAC program [24].

### 2.3. Gridless Generalized Docking and Low-Energy Minima Sets

Until recently most of the docking programs used a grid of precalculated energy potentials of probe ligand atoms in the field of target protein atoms. These potentials included Coulomb and van der Waals interactions and sometimes partial interaction with solvent (see, e.g., [30, 31]). This approach obviously cannot take into account mobility of protein atoms. It is also impossible to carry out the local energy optimization with variations of coordinates of ligand and protein atoms and to take into account solvent in one of implicit (continuum) solvent models, since solute atoms charges interaction with polarized charges induced on the surface of dielectric continuum surrounding the solute molecule are the nonlocal effect, and this cannot be carefully represented by local potentials in the grid nodes. Therefore, programs for gridless generalized docking [1–3] have been developed recently to increase the docking accuracy. These programs do not calculate the grid of potentials in advance. The energy of the protein-ligand complex for any given configuration is computed directly with the MMFF94 force field without any fitting parameters. Also, it is possible to consider solvent in one of implicit solvent models in these programs.

Generalized docking with the new gridless FLM docking program was carried out for 16 test protein-ligand complexes [1, 2]. The improvement of ligand positioning was found when the energy target function in vacuum was replaced by the energy function in solvent. In addition the improvement of ligand positioning was found when the quantum chemical PM7 method for protein-ligand energy calculation was used instead of the classical MMFF94 force field. Low-energy minima spectra of test protein-ligand complexes were found in the docking procedure when about half a million local optimizations were performed for one native ligand docking. It was shown that the best positioning accuracy was achieved for the energy function calculated with the MMFF94 force field and the PCM (the Polarized Continuum Model) implicit solvent model [28, 29]. For this energy function 8192 low-energy minima including the global one were found and stored for each protein-ligand complex. This set of minima was designated as {2}MMFF94 + PCM in works [1, 2]. Just these minima are used in the present study as an initial source for the energy recalculation by the MOPAC [24] program with the PM7 method [5] and the COSMO solvent model [9]. The local optimization of the energy of the protein-ligand complex is performed from the initial configuration corresponding to each minimum of the minima set {2}MMFF94 + PCM using the PM7 method [5] in vacuum with variations of all ligand atoms Cartesian coordinates. Then the energy of the protein-ligand complex corresponding to each newly found minimum is recalculated taking into account the COSMO solvent model. The resulting new minima set is designated as {2}PM7 + COSMO and its minima are ranked in order of their energies. For most test complexes the PM7 energy optimization is successful for ~8000 minima, but the optimization of the 1c5y complex is successful for only ~2500 minima.

Also, energies of all 8192 minima of the {2}MMFF94 + PCM set are recalculated without geometry optimization (1SCF routine of the MOPAC program) using the PM7 method with the COSMO solvent model, and then they are ranked in order of their energies and this minima's set is designated as {2}PM7 + COSMO (1SCF).

The native ligand is locally optimized from its initial crystallized position using either the MMFF94 force field or the PM7 method in vacuum, and then the energy of the minimum is recalculated with the PCM or the COSMO solvent model, respectively.

## 3. Results and Discussion

All found local energy minima of a given protein-ligand complex for a given target energy function can be sorted by their energies in the ascending order; that is, every minimum gets its own index equal to its number in this sorted list of minima. The lowest energy minimum has index equal to 1. We introduce two types of minima indexes [1–3] to analyze the feasibility of the docking paradigm as follows.

The list of minima can include some minima corresponding to the ligand position located close to the nonoptimized native (crystallized) position of the ligand, that is, located close to the position of the ligand in the given protein-ligand complex atomic structure taken from the Protein Data Bank database [32]. We consider that the ligand is in close proximity to the nonoptimized native ligand position if the RMSD (the root mean square deviation, between equivalent atoms of the ligand in the two positions) is less than 2 Å. Let us designate the index of such energy minimum which is close to the native (crystallized) ligand position as INN (it is the abbreviation of “Index of Near Native”). If there are several such minima which are close to the crystallized ligand position, we choose the minimum with the lowest energy (with the lowest index) as “INN.” Further, if we include the locally optimized native ligand position into the list of minima ranked by their energies, such optimized native ligand minimum also gets some index. We designate this index as IN (Index of Native). If IN = 1 and INN = 1, we can say that the docking paradigm is true: the lowest found energy minimum, that is, the global energy minimum, is close to the crystallized (native) position of the ligand, and also this crystallized ligand being locally optimized has the lowest energy among energies of all other minima. Obviously, if the docking program or quasi-docking program finds the set of low-energy minima with the indices IN = 1 and INN = 1, then the positioning accuracy can be considered as excellent. In this case we can be sure that the global energy minimum corresponds either to the locally optimized native (crystallized) ligand position or to the ligand pose close to the native (crystallized) ligand position.

Values of IN and INN indexes are presented in Table 1 for different energy minima sets for all tested protein-ligand complexes.

It can be seen from Table 1 that the energy recalculation with the PM7 method leads to the appreciable decrease of IN and INN indexes. For example, IN/INN indexes of the 4fta complex become equal to 2/1 instead of 186/187; that is, both indexes decrease almost to their minimal values. This situation takes place for almost all tested complexes even without the additional optimization of the ligand position with the PM7 method. This means that the global energy minimum is close to the native (crystallized) ligand position (INN = 1) when the protein-ligand energy is calculated with the PM7 method and solvent in the COSMO model. At the same time the minimum obtained by the ligand local optimization from its initial crystallized position has the lowest (IN = 1) or almost the lowest energy (IN = 2–5).

So, the docking paradigm is true for almost all tested complexes when minima energies are calculated by the quantum chemical method. There is some worsening of indexes; that is, the increase of IN and INN indexes, for some complexes when the PM7 energy optimization is performed: indexes for 4fsw, 1f5l, and 1vj9 complexes in the 3rd and 4th columns of Table 1 have to be compared. This finding can probably be associated with the following reasons: (i) the incompleteness of the {2}MMFF94 + PCM minima set used as initial configurations for PM7 energy calculations (e.g., the native ligand from the 1vj9 complex has 19 torsions and it is too flexible), (ii) nonoptimal geometries of the target proteins when the PM7 energy optimization was performed (each protein was taken from PDB [32] as it is with only hydrogen atoms incorporation by the APLITE program [31]), and (iii) faults of the PM7 method [27, 33]. The first possible reason (i) could be avoided by the choice of more appropriate test complexes. The second reason (ii) could be resolved by some preliminary protein optimization with the PM7 method [34], and faults of the PM7 method (iii) could be possibly avoided by using the PM6-D3H4(X) method.

Similar strategy as in the current work has been recently used in the newly defined scoring function (the SQM/COSMO filter) on the base of the PM6-D3H4X semiempirical method and the COSMO solvent model [6], and a superior performance of this scoring function has been demonstrated in comparison with eight statistics-, knowledge-, or force field-based scoring functions using two criteria, the number of false positives and the maximum RMSD from the native (crystalized) ligand pose within a defined interval of the normalized score. Better positioning accuracy with the PM7 semiempirical energy target function comparing with MMFF94 one is also demonstrated in the current work for the wider range of protein-ligand complexes. However, in the present work the initial minima set has been obtained in the docking procedure [1, 2] with MMFF94/PCM energy calculations without any fitting parameters (in contrast to docking with Glide, PLANTS, Autodock Vina, and GOLD in [6]) and we can make more rigorous comparison of the docking performance with the MMFF94 force field and with the PM7 method. In addition, in the present work we use a much larger set of initial minima (~8000) for each complex than in the work [6] (2865 poses in total; and not all of these poses were minima for the respective scoring function). Our observations show that after energy recalculation with the semiempirical method the order of minima energies can change dramatically comparing with the force field energies and the more minima are stored the better results on the positioning accuracy are obtained. We also did not use any postprocessing as it was made with AMBER/GB in [6] when hydrogen atoms and close contacts were relaxed.

## 4. Conclusions

The present study demonstrates the significant improvement of the ligands positioning accuracy and the feasibility of the docking paradigm with the sequential use of MMFF94 force field docking and the subsequent recalculation of minima energies with the PM7 quantum chemical method. The implicit solvent models are very important in both cases. These conclusions support clearly previously reported results for the same force field and semiempirical method [1, 2] and for another semiempirical method (PM6-D3H4X) with different force fields scoring functions [6].

This result shows that the future rigorous docking when the whole low-energy minima spectrum of the protein-ligand complex will be found with the PM7 or with another quantum chemical method, for example, PM6-D3H4X, should improve considerably the accuracy of ligand positioning and consequently improve the accuracy of protein-ligand binding energy calculations. Undoubtedly, the fast increase of available supercomputer resources creates possibility of performing rigorous PM7 quantum chemical docking in the next 1-2 years. Such docking will create the base for a substantial improvement of effectiveness of the docking application for the new drugs development. We used more than 10 million CPU-hours to conduct these quasi-docking calculations, and these investigations became possible only due to computing resources of M.V. Lomonosov Moscow State University supercomputer Lomonosov [35].

## Figures and Tables

**Table 1:** IN/INN indexes for minima sets: {2}MMFF94 + PCM, {2}PM7 + COSMO (1SCF), and {2}PM7 + COSMO. Complex ID identifies the respective protein-ligand structure in the Protein Data Bank [32]. {2}MMFF94 + PCM designates the low energy minima set found in works [1, 2] for the target energy function calculated in MMFF94 force field with the implicit PCM solvent model (8192 low energy minima). Energies of all 8192 minima of the {2}MMFF94 + PCM set are recalculated without geometry optimization using the PM7 method with the COSMO solvent model, and this minima set is designated as {2}PM7 + COSMO (1SCF). The low energy minima set designated as {2}PM7 + COSMO is obtained by the local optimization of the energy of the protein-ligand complex from the initial configurations corresponding to each minimum of the minima set {2}MMFF94 + PCM using the PM7 method in vacuum with variations of all ligand atoms Cartesian coordinates and subsequent recalculation of minima energies taking into account the COSMO solvent model.

Complex ID	{2}MMFF94 + PCM	{2}PM7 + COSMO	{2}PM7 + COSMO
		1SCF	
4ft0	164/159	2/1	2/1
4ft9	3/1	2/1	1/1
4fsw	134/140	2/1	112/80
4fta	186/187	2/1	2/1
4fv5	6/3	1/1	2/1
4fv6	3/68	1/1	1/1
1dwc	250/35	5/2	9/4
1tom	13/4	2/1	6/1
1c5y	2/1	1/1	19/1
1f5l	10/1	2/2	92/39
1o3p	274/1	54/1	17/7
1sqo	54/1	2/1	5/1
1vj9	11/18	2/14	1/74
1vja	1/2	2/2	1/4
2p94	35/1	1/1	5/1
3cen	35/1	3/1	65/1
